# Systematic review and meta-analysis of tick-borne disease risk factors in residential yards, neighborhoods, and beyond

**DOI:** 10.1186/s12879-019-4484-3

**Published:** 2019-10-17

**Authors:** Ilya R. Fischhoff, Sarah E. Bowden, Felicia Keesing, Richard S. Ostfeld

**Affiliations:** 1 Cary Institute of Ecosystem Studies, 2801 Sharon Turnpike, Millbrook, NY 12545 USA; 2 Eagle Medical Services, LLC, 2835 Brandywine Rd. Suite 200, Atlanta, GA 30341 USA; 3 Division of Global Migration and Quarantine, Centers for Disease Control, 1600 Clifton Road, Atlanta, GA 30329-4027 USA; 4 Bard College, PO Box 5000, Annandale-on-Hudson, New York, 12504 USA

**Keywords:** Peri-domestic, *Ixodes scapularis*, Lyme disease, Babesiosis, Anaplasmosis, Tick bites, Spatial scale

## Abstract

**Background:**

Exposure to blacklegged ticks *Ixodes scapularis* that transmit pathogens is thought to occur peri-domestically. However, the locations where people most frequently encounter infected ticks are not well characterized, leading to mixed messages from public health officials about where risk is highest.

**Methods:**

We conducted a systematic review and meta-analysis on spatial risk factors for tick-borne disease and tick bites in eastern North America. We examined three scales: the residential yard, the neighborhood surrounding (but not including) the yard, and outside the neighborhood. Nineteen eligible studies represented 2741 cases of tick-borne illness and 1447 tick bites. Using random effects models, we derived pooled odds ratio (OR) estimates.

**Results:**

The meta-analysis revealed significant disease risk factors at the scale of the yard (OR 2.60 95% CI 1.96 – 3.46), the neighborhood (OR 4.08 95% CI 2.49 – 6.68), and outside the neighborhood (OR 2.03 95% CI 1.59 – 2.59). Although significant risk exists at each scale, neighborhood scale risk factors best explained disease exposure. Analysis of variance revealed risk at the neighborhood scale was 57% greater than risk at the yard scale and 101% greater than risk outside the neighborhood.

**Conclusions:**

This analysis emphasizes the importance of understanding and reducing tick-borne disease risk at the neighborhood scale. Risk-reducing interventions applied at each scale could be effective, but interventions applied at the neighborhood scale are most likely to protect human health.

**Trial registration:**

The study was registered with PROSPERO: CRD42017079169.

## Background

An estimated 300,000 cases of Lyme disease occur annually in the United States [1, 2], and the geographic range of Lyme disease in North America is rapidly expanding [3]. Throughout most of North America, the causative agent of Lyme disease, the bacterium *Borrelia burgdorferi* sensu stricto, is transmitted by the blacklegged tick *Ixodes scapularis* [1]. *I. scapularis* also transmits other important zoonotic pathogens, including the bacterium that causes anaplasmosis, the protozoan that causes babesiosis, and Powassan virus.

Exposure to infected blacklegged ticks is thought to occur primarily peri-domestically [4–7], which has led to widespread interest in deploying methods for controlling ticks and avoiding exposures in residential yards [8]. However, the evidence is equivocal for the assertion that human-tick encounters responsible for zoonotic transmission events occur predominantly around the home. In one study of 70 people bitten by ticks in Westchester County, NY, 69% reported acquiring the tick in their backyard, with the remaining ticks being acquired at school or camp (11%), in parks or recreational areas (9%), at work (4%), while hunting (3%), or elsewhere (4%) [9]. In Connecticut, ticks (*N* = 4717 records) submitted to the state health department included 74% self-reported as being acquired outside at home, 5% acquired in the neighborhood, and 21% away from home [10]. In one Rhode Island community, households with Lyme disease cases had higher density of infected nymphal ticks in their yards than did households without Lyme disease cases, yet this “entomologic risk” was not a significant predictor of Lyme disease over 2 years, suggesting that additional factors such as human behavior played a role [4]. A randomized, controlled study in Connecticut, New York, and Maryland found that tick abundance was lower in yards treated with the acaricide bifenthrin, compared to yards sprayed with a placebo. Nonetheless, there was no significant difference between participants with bifenthrin-treated versus placebo-treated yards in the frequency of tick bites or tick-borne diseases [11]. Possible explanations for these results include participants having encountered ticks outside their yards, or in parts of their yards that were not sprayed (including the interior of wooded areas, vegetable gardens, and flower beds) [11].

Quantifying the relative contribution of yard-level risk factors, versus risks associated with factors at other scales, is important for public health. Some public health officials recommend landscaping yards [12] and using acaricides in yards [13, 14] to reduce disease risk, at a significant financial and possible environmental cost. In a survey of 1200 residents in three Connecticut towns, 31% of respondents used acaricides in their yards [15]. People also invest in community-wide efforts to reduce risk, including deer hunts and deployment of deer-targeted acaricides such as “four-posters” [16]. In addition to informing decisions by households and communities about where to focus tick control efforts, understanding where people are at risk is also important in deciding where to employ behaviors intended to prevent tick bites, such as use of repellent, protective clothing, or body checks after time spent outdoors.

Increases in the frequency of tick-borne diseases highlight the importance of a systematic review and meta-analysis of the spatial component to risk of tick-borne disease in eastern North America, where Lyme disease has been endemic for decades. We classified potential risk factors spatially as relating to the yard (peri-domestic) [5, 17, 18], the neighborhood surrounding but not including the yard, and areas outside the neighborhood. We compared the relative magnitudes of risk factors associated with these spatial scales. The scales are defined to be mutually exclusive, to enable drawing conclusions from the meta-analysis about the scale or scales at which interventions are expected to be most effective. These three spatial scales are relevant to decisions of individuals and communities. Public health messages, interventions, and decision-making processes may differ depending on whether the aim is actions in individual yards, across a neighborhood, or at the scale of a larger region extending beyond the neighborhood.

## Methods

### Search strategy and selection criteria

We performed a systematic review and meta-analysis, following Preferred Reporting Items for Systematic Reviews and Meta-Analyses (PRISMA) guidelines (Additional file 1). The study protocol was registered in the PROSPERO Register CRD42017079169 (Prospective International Register of Systematic Reviews; www.crd.york.ac.uk/prospero) at the beginning of the study. We found an initial set of six articles using a series of topic searches in Web of Science, including the terms “peridomestic AND ticks”; “yard AND ticks AND bite”; “tick AND encounter AND risk”; and “Lyme disease AND case-control” [4, 17–21]. We identified risk factors in those six articles (Additional file 2). We used these risk factors as keywords in a more comprehensive Web of Science search (Additional file 3). We added terms on variables at neighborhood and greater scales. We restricted search terms to articles on diseases caused by pathogens transmitted by *I. scapularis*, in U.S. states and Canadian provinces with recent Lyme disease incidence of at least 1 per 100,000 [22, 23]. Due to resource constraints, we did not search other databases besides Web of Science.

We defined the yard as the area within a household’s property boundaries. We defined the neighborhood as outside the yard but within 500 m of the boundary. This distance is commonly used in defining neighborhoods, and encompasses typical walking distances from home to locations such as banks, supermarkets, and post offices ([24]; [25]). Places further than 500 m from a property were defined as outside the neighborhood. From the perspective of a focal person living in a neighborhood, the neighborhood will typically include other people’s yards, which are peri-domestic for the people living in those neighboring properties but not peri-domestic for the focal person. When a person visits neighbors’ yards, or other places in the neighborhood, risks from ticks may be different than when the person is in their own yard, for example due to differences in human behavior around the home versus elsewhere. Neighborhood-scale factors, such as habitat of areas adjacent to the yard or vertebrate hosts that move across property boundaries, may influence risk within the yard. We define each spatial scale to exclude the other two, in order to enable analyses that identify the spatial scales at which public health interventions are likely to have greatest impact. We assigned spatial scales to the risk factors in the initial set of six articles (Additional file 2: Table S1). In many cases, although a study did not specify where an activity took place, it was possible to make a reasonable judgment, for example that camping or outdoor occupations typically took individuals away from their yard and neighborhood. For certain activities, such as hunting and fishing, surveys indicate 79% of participants travel a distance greater than five miles (eight km) to reach their recreational destination [26]. For other recreational activities, such as hiking or camping, we are not aware of direct sources of information on travel distances, however distances from towns to parks (common destinations for these recreational activities) suggest hiking or camping typically takes visitors outside the neighborhood [27]. We incorporated data on self-protective behaviors, such as use of repellent or self-checks for ticks, where studies specified where that activity took place. Occupational exposure we assumed to be outside the neighborhood. We excluded variables such as “hours spent in vegetation”, “own a dog”, or “rural residence” that we could not reliably assign to a spatial category. We further excluded activities such as “walk or jog outdoors” [21] in which people’s movements frequently span the neighborhood to outside the neighborhood.

We performed the full search of Web of Science on July 30, 2017. For an initial 50% of abstracts (619 randomly selected among the total 1237), one author (IRF) carried out full-text eligibility assessment for abstracts relating spatial risk factors to disease incidence as well as case-control data. Of the 619 abstracts screened, 20 concerned disease incidence (Additional file 4), and these were subjected to detailed examination. Each incidence article reported risk factors at spatial scales approximating a neighborhood (e.g., census block group [28], ZIP code [29]), were at greater-than-neighborhood scales (e.g., municipality [6], county [30]), or were at variable scales (areas bounded by U.S. federal roads [31]). As these incidence studies did not enable comparison of relative risks across the three spatial scales, we excluded incidence studies from the systematic review and meta-analysis.

**Fig. 1:**
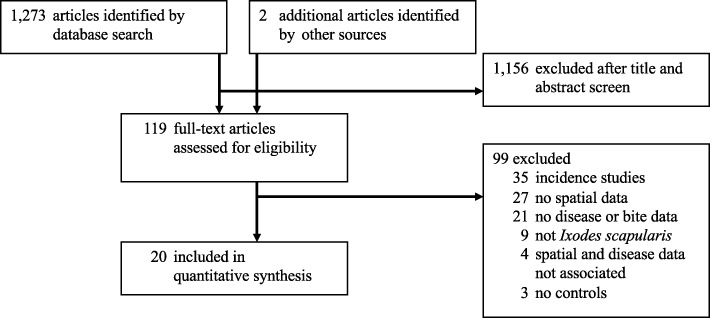
Preferred Reporting Items for Systematic Reviews and Meta-Analyses diagram

Following the initial assessment of incidence studies, we screened each article’s title and abstract, without seeing any other article information. We evaluated the full articles for abstracts reporting on tick bites or tick-borne disease case-control, longitudinal, controlled or non-controlled trials, or survey studies including risk factors at one or more relevant spatial scales. Two people screened each abstract and title. If at least one screener determined that an abstract was relevant, then we assessed the article in full. If the article reported data that we could code to spatial scale, in association with disease case-control or odds ratio data, then we included it in the systematic review and meta-analysis. In the case of studies on tick bites or ticks found crawling on a person, we included the study if it reported spatial data in association with data on individuals who were bitten versus those who were not, or indices of exposure (e.g., anti-tick salivary antigen) [32]. Studies were included regardless of the criteria by which studies defined cases of tick-borne disease. In addition to excluding incidence articles, we also excluded reviews, articles on ticks other than *I. scapularis*, articles in which spatial data were dissociated from case-control data, and articles lacking spatial data, case or bite data, or controls. Figure 1 illustrates the screening and review steps, and the numbers of articles at each step.

There are several aspects of meta-analyses that require the results of our study to be interpreted with caution, but that we do not expect to have introduced systematic biases. As with all meta-analyses, ours can only include effects measured in the original papers. We relied on each paper’s descriptions of the spatial scale associated with risk factors. While we recognize that factors reported at one spatial scale may suggest conditions or behavior at other scales, we did not assume this to be the case. If, for instance, a paper provided data on study participants’ use of a self-protective measure, such as use of repellent, while study participants were in the yard, we classified this as a yard-scale data point but did not assume that participants’ adherence to this measure extended to other spatial scales unless the paper specified as much. Studies did not provide information on relative exposure to ticks at each scale, thus it was not possible to account for effects of potential differences in exposure by scale. We are further unable to incorporate latent spatial variables, such as landscape structure or vertebrate host community, that may have effects on risk but were not included across all the original papers at all scales. We do not expect these limitations of meta-analysis to present biases, because we expect researchers have examined and reported on factors they consider to be of importance.

### Data analysis

**Fig. 2:**
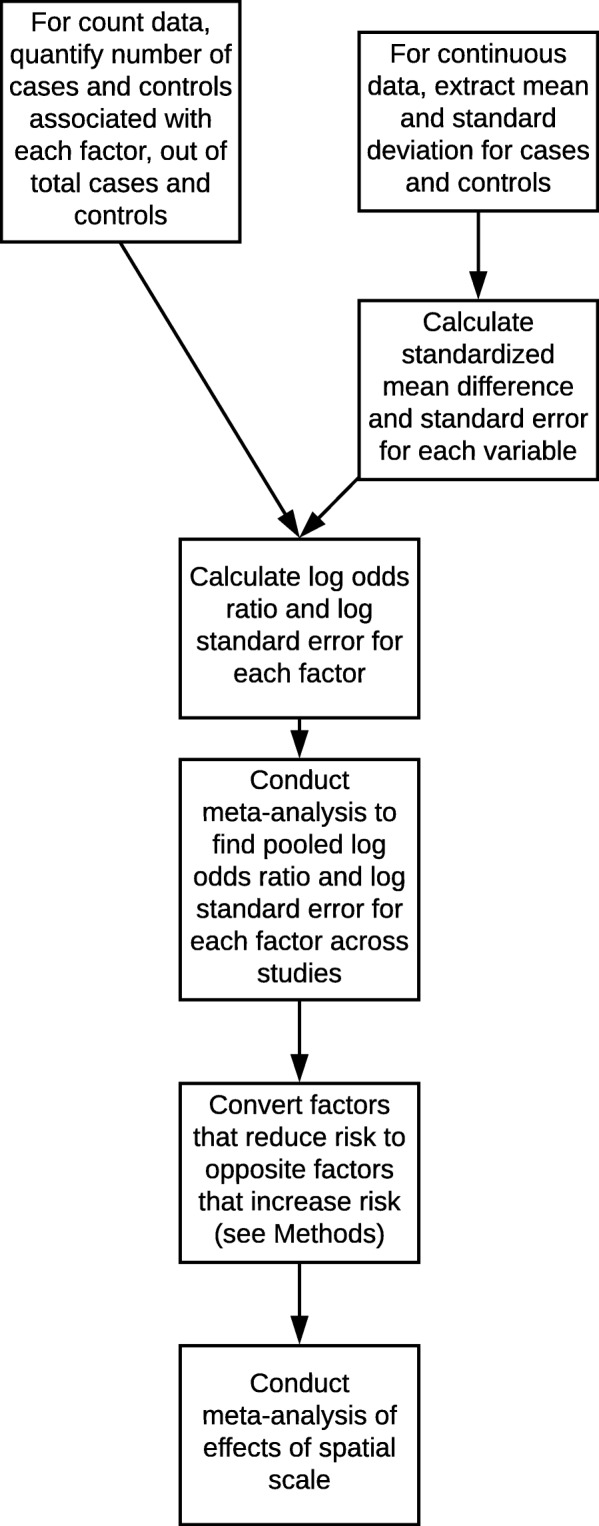
Flow-chart of conversion of multiple data types into common format and use in meta-analyses

Studies reported results in several ways, including numbers of individuals associated with a potential risk factor or percent of individuals, numbers of cases and controls or odds ratios computed from numbers of cases and controls, and numbers of individuals associated with a variable versus continuous variables measured for individuals. We converted this range of data types into uniform data types, log odds ratios and log standard errors ratios, which we then used in meta-analyses. Figure 2 depicts a flow chart for the steps of processing a range of data types for meta-analysis.

For studies that reported numbers of tick-borne disease or tick exposure cases and controls associated with risk factors, we used these data to compute the log odds ratio and log standard error for each risk factor. We computed these values using the Mantel-Haenszel method via function “rma.mh” in R package “metafor” [33]. We conducted all analyses in R version 3.4.0 [34]. For the controlled trial studies, we analyzed the reported frequency of cases and controls in relation to the risk factors. If a study reported the percent of cases and controls associated with a variable, we converted percent values to numbers of individuals and then computed the log odds values. Where a study provided odds ratios and 95% confidence intervals, we converted the odds ratio into the log odds ratio and computed the log standard error using the confidence intervals: *logSE* = (*logCI*(*Upper Bound*) −  *log* (*Odds Ratio*)) /1.96 [35].

For studies that reported continuous variables, e.g., mean weekly outdoor work hours for cases and controls [36], we first computed the standard mean difference *d*, between cases and controls, and the standard error around *d*, *SE*_*d*_ using function “escalc” in package “metafor” [33]. Then we converted from *d* to the log odds ratio: $Log(OddsRatio)=d\frac{\pi }{\sqrt{3}}$, and from *SE*_*d*_ to the log standard error: $\log (SE)= SE(d)\frac{\pi }{\sqrt{3}}$ [37].

Studies reported data for each risk factor separately, without including raw, individual-level data or cross-correlations among factors. We therefore analyze each risk factor as independent of others within each study, without including potential cross-correlations among risk factors. Some studies reported odds ratios and confidence intervals that had been adjusted for demography or other variables [36]; in these cases we used these adjusted values ratio to compute the log odds ratio and log standard error. Considering studies that reported case-control data for multiple levels of a variable within the same spatial category, we used only the measure with the highest reported risk, to avoid pseudoreplication.

Certain factors may increase risk while others decrease risk. In order to compare the relative magnitudes of factors that increase risk across spatial scales, we converted factors that reduce risk into the converse factors that increase risk. We first reclassified for consistency near-synonymous variables described using different terms across studies (e.g., reclassifying as “hunting” studies that described “hunt” vs. “hunting”). Using the “rma.uni” function in package “metafor” [33], we constructed a linear mixed model to test for the effect of the specific variables measured in studies (e.g., “bird feeder”; “woods”; “fishing”). Study was a random effect. Based on the fitted model, we found the estimated mean log odds value for each specific variable across all studies and spatial categories, including variables associated with both disease and tick bites (see Additional file 5 for log odds ratio estimates for each specific variable). If the average log odds ratio was negative for a variable, indicating that the variable may reduce risk, then we used the converse variable and multiplied the log odds ratio from each study for that variable by negative one. For example, the estimated log odds ratio for “use of repellent” was negative (indicating reduced risk associated with repellent use), therefore we multiplied the reported values for this variable by negative one in each study, and analyzed the converse variable, “not using repellent”, in all further analyses. Similarly, having a fence on one’s property was associated with negative log odds values, so we multiplied the log odds ratio values for this variable by negative one and analyzed the variable “not having a fence”. Out of 64 variables, we undertook this conversion for the 16 variables that had a negative pooled log odds ratio value.

### Statistical modeling

Sample size was not adequate to permit accounting for study location in the analysis of effects of spatial scale. However, we evaluated whether study location correlated with disease risk and therefore represented a potential confound. We determined whether there was a relationship between Lyme disease incidence in study areas and the frequency of measures in each spatial scale. For each study, we found the average Lyme disease incidence over the period 2006 to 2016 for the state in which the study was conducted [22]. For studies that spanned multiple states we took the average incidence across those states. For the study in Canada [38], we assigned the mean Lyme disease incidence in Canada over the period 2009-2016 [39]. Risk factors for disease were assigned to one of four categories, corresponding to the Lyme disease incidence of the study area relative to the quartiles of the distribution of Lyme disease incidence values for U.S. states and Canada. To evaluate the potential relationship between Lyme disease incidence quartile and the frequency of observations at each spatial scale, we applied a Chi-square test. We used function “chisq.test” in R “stats” package, with simulated P values to accommodate low counts at the neighborhood scale.

To determine the appropriate model formulation for testing for effects of spatial scale, we first constructed a series of alternative linear mixed models using the following predictors: spatial scale, publication year (relative to the year of the first study published), or both spatial scale and publication year. We included publication year as a predictor based on exploratory analysis that indicated a potential decreasing trend in log odds ratios in relation to publication year. Study was a random effect in each model. We used the “rma.uni” function in R package “metafor” (Viechtbauer, 2010). We compared the fit of alternative models using the Akaike Information Criterion for small samples, AICc, with the R package “AICcmodavg” [40, 41]. We considered models with *∆AICc* < 2 (the difference between a model’s *AICc* value and the *AICc* value of the best-fitting model) to have a similar level of support [40].

Based on the result (Table 1) that the best fitting model included both spatial scale and publication year, we proceeded to include both of these variables in our analyses of disease risk. For tick bites, there was only one measure at the neighborhood scale, below the minimum of two observations for a meaningful pooled estimate at this scale [42]; thus we limited the analysis to the scales of the yard and outside the neighborhood. There remained only 11 tick bite measures across all studies, resulting in low statistical power to detect effects of both spatial scale and publication year [43]; therefore, we tested for effects of spatial scale only. We did not incorporate study quality in the meta-analysis, due to the lack of objective criteria for weighting these studies by quality.

**Table 1:** Comparison of alternative models for risk of tick-borne disease or tick bites. The best-fitting model included effects of spatial scale and publication year

Model terms	AICc	delta AICc	Likelihood	AIC weight
Space + year	148.8	0	1	1
Space	166	17.2	0	0
Year	192.1	43.3	0	0

For the best-fitting model of disease, we used analysis of variance to compare pairs of means at the different spatial scales, using function “anova” in the R stats package. We analyzed log odds ratios, but present results as odds ratios, as the linear scale is more readily interpretable. Data files are available [44].

## Results

From 1237 citations, 19 studies met the criteria for inclusion; we also reviewed two additional studies that were identified by reviewers, and included one of these in the meta-analysis [45]. In all, the 20 studies provided 98 estimates of disease risk and 12 estimates of tick bite risk (Additional file 6). The total study population included 2741 individuals with tick-borne diseases and 1447 individuals who experienced tick bites or ticks crawling on them.

The studies took place in 14 U.S. states and in Canada. Based on the Chi-square test, we detected no significant relationship (χ^2^ = 11.007, *P* = 0.0949; Additional file 7) between Lyme disease incidence in the study location (by quartile of the incidence distribution) and counts of observations of disease risk factors across studies at each spatial scale.

**Fig. 3:**
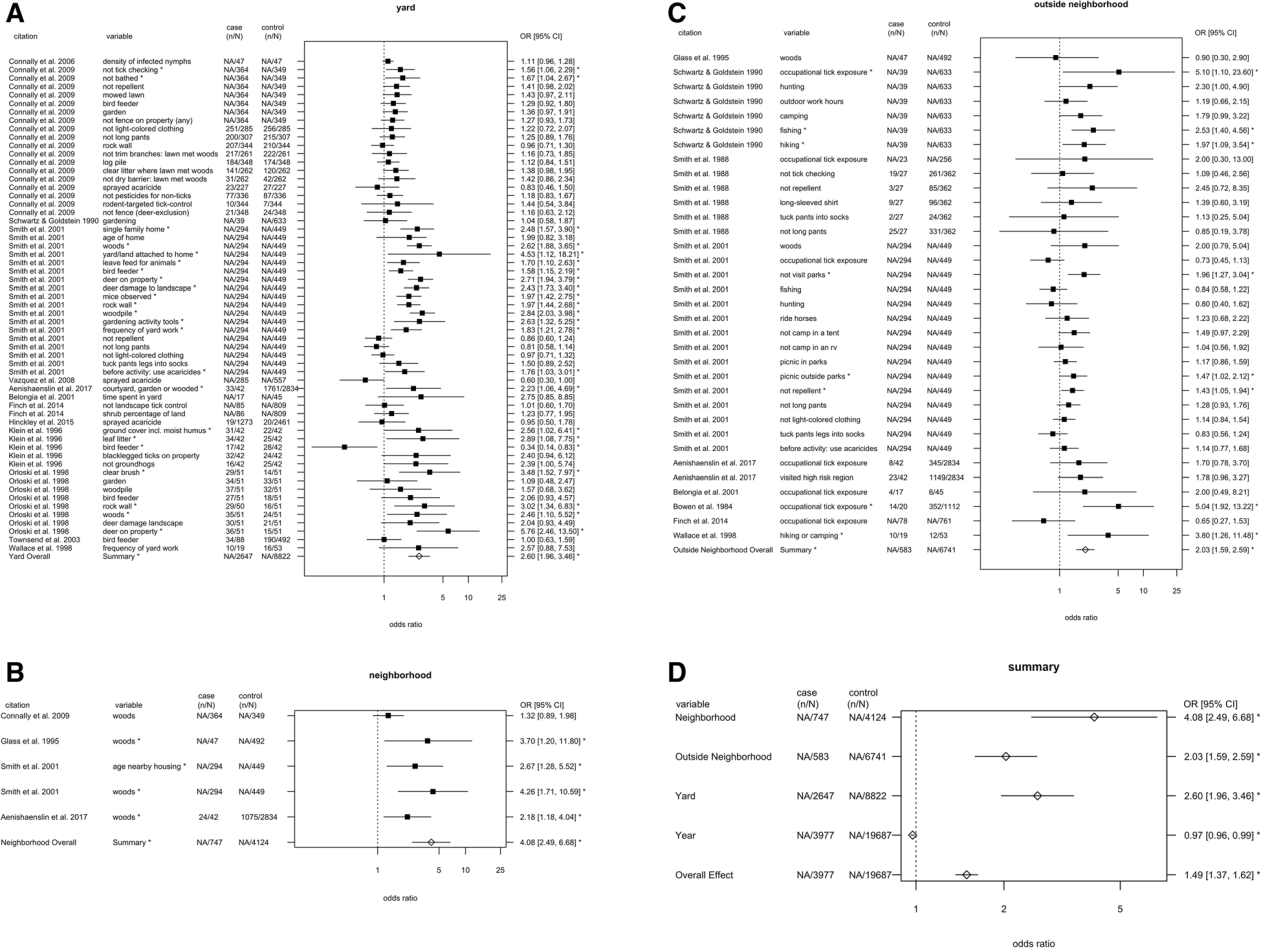
Odds ratios with 95% confidence intervals for estimates of tick-borne disease risk associated with variables at the scale of the (**a**) yard; (**b**) neighborhood; (**c**) outside the neighborhood; and (**d**) overall effects. Self-protective behaviors such as use of repellent were assigned to spatial scale based on descriptions in the original articles of where individuals performed these activities (e.g. “before working or playing in yard: apply insect repellents” [21]). Case (n/N) refers to the number of individuals with a risk factor (n) out of the total number with a tick-borne disease (N); control (n/N) refers to the number with a risk factor out of the total without a tick-borne disease. NA typically indicates that the odds ratio was used rather than the count. The pooled estimates included fixed effects of publication year and random effects of study. The overall effect is from a model that included only the random effects. Filled squares indicates estimates from a single study; open diamonds are overall effects. Asterisks denote estimates for which the 95% confidence interval around the odds ratio excluded one

Model comparison indicated that the best-fitting model included effects of spatial scale and publication year (Table 1). For disease risk, there were significant effects of spatial scale, at each scale: the yard, neighborhood, and outside the neighborhood (Table 2, Fig. 3). In addition, there was an effect of publication year, with risk declining modestly over time (odds ratio = 0.97, *P* < 0.0001). Risk at the neighborhood scale was 57% greater than at the yard scale and 101% greater than outside the neighborhood, while risk at the yard scale was 28% greater than outside the neighborhood (Table 3). The *I*^2^ value for residual heterogeneity was 57%, indicating that the model accounted for 43% of the variation in risk. The test for residual heterogeneity was significant (QE = 216.0, df = 94, *p* < 0.0001), indicating that other factors not included in the model may have affected risk.

**Table 2:** Estimated odds ratio, standard error, 95% confidence interval, and *P*-values for effects of spatial scale and publication year on tick-borne disease risk. There were significant effects of scale and year (Wald test statistic [df = 4] = 117.6, *P* < 0.0001)

Spatial scale	Odds ratio	SE	CI	*P*	Number of studies	Number of records
Yard	2.60	1.16	[1.96, 3.46]	<0.0001	13	59
Neighborhood	4.08	1.29	[2.49, 6.68]	<0.0001	4	5
Outside Neighborhood	2.03	1.13	[1.59, 2.59]	<0.0001	9	34
Year	0.97	1.01	[0.96, 0.99]	0.0001	NA	NA

**Table 3:** Pairwise comparisons of log odds ratios for yard, neighborhood and outside neighborhood spatial scales. The pairwise comparisons result from analysis of variance of the fitted linear mixed effects model, which included fixed effects of spatial scale and publication year and random effect of publication. Differences are the value for the first spatial scale, minus the value for the second scale (e.g., neighborhood minus outside neighborhood). Ratios are the odds ratio for the first spatial scale, divided by the odds ratio for the second spatial scale

Comparison	Difference in log odds ratios	Standard error of estimated difference	Z value	*P* value	Ratio of odds ratios
Neighborhood versus outside neighborhood	0.7	0.22	−3.17	0.002	2.01
Neighborhood versus yard	0.45	0.21	−2.16	0.03	1.57
Yard versus outside neighborhood	0.25	0.1	2.61	0.009	1.28

**Fig. 4:**
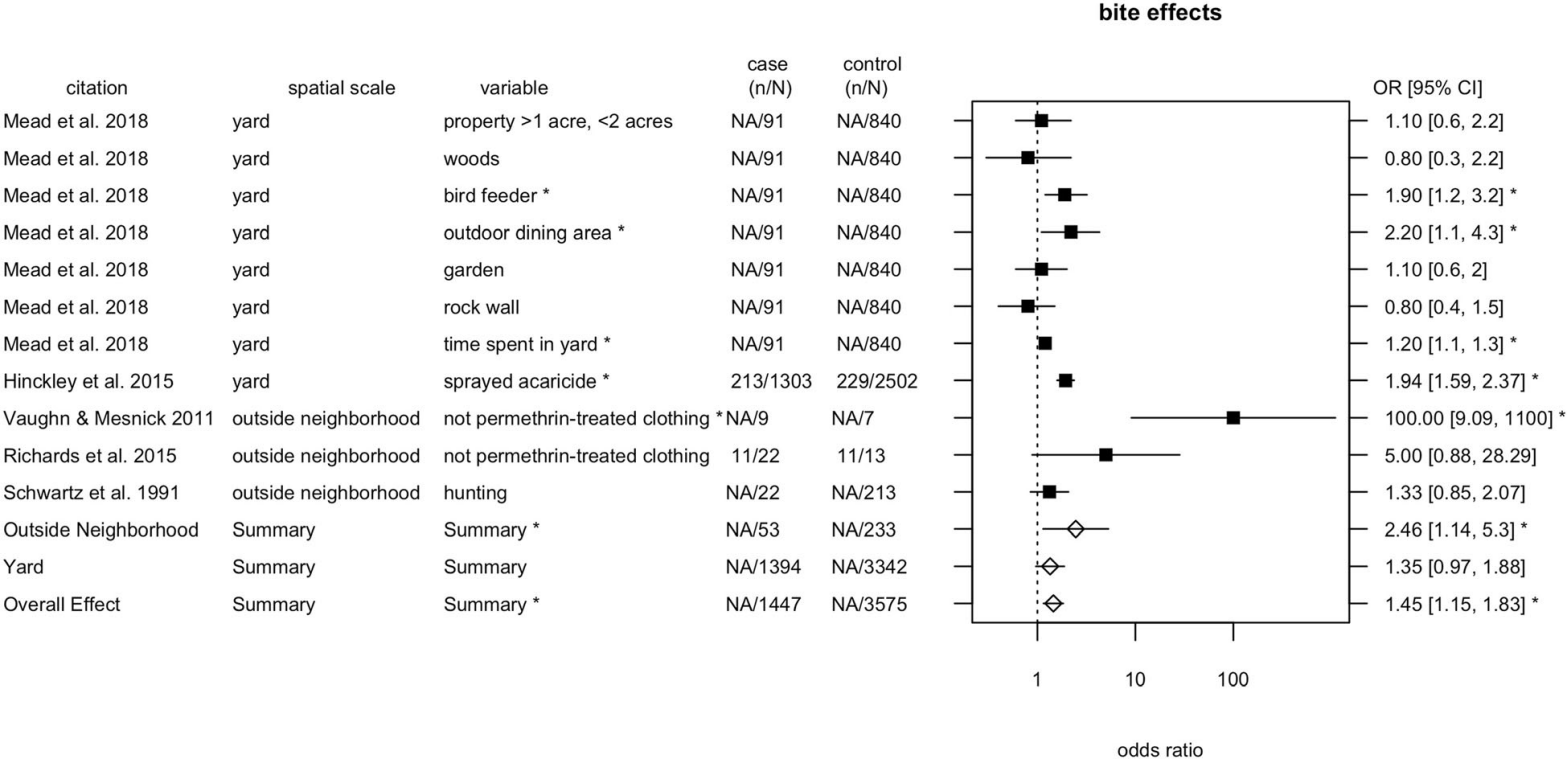
Odds ratios with 95% confidence intervals for tick bite risk factors. The meta-analysis included fixed effects of spatial scale and random effects of study. Case (n/N) refers to the number of individuals with a variable (n) out of the total number with a bite (N); control (n/N) refers to the number with a risk factor out of the total without a tick-borne disease. Filled squares indicates estimates from a single study; open diamonds are overall effects. Asterisks denote estimates for which the 95% confidence interval around the odds ratio excluded one

For tick bites, we detected a significant effect of spatial scale, at the scale of outside the neighborhood (Table 4, Fig. 4). The *I*^2^ value was 82%, indicating that the model accounted for 18% of the variation. The test for residual heterogeneity was significant (QE = 40.94, df = 9, *p* < 0.0001), indicating that not all factors affecting risk were captured by the model. The neighborhood scale was excluded from the meta-analysis because there was only one observation at this scale. The data point at the neighborhood scale was time spent in someone else’s yard (odds ratio = 1.1, 95% CI = [1.0, 1.3]) [45].

**Table 4:** Estimated odds ratio, standard error, 95% confidence interval, and *P*-values for effects of spatial scale on tick bite risk. The linear model included a random effect of publication. There was a significant effect of spatial scale (Wald test statistic [df = 2] = 8.36, *P* = 0.0152)

Spatial scale	Odds ratio	SE	CI	*P*	Number of studies	Number of records
Yard	1.35	1.19	[0.97, 1.88]	0.0799	2	8
Outside neighborhood	2.46	1.48	[1.14, 5.3]	0.0213	3	3

## Discussion

Exposure to infected blacklegged ticks is thought to occur peri-domestically, but rigorous assessment of this hypothesis has been lacking. Indeed, the locations where people encounter infected *Ixodes scapularis* ticks have been identified as an important unresolved issue in understanding tick-borne disease risk [46]. Tick-bite self-reports suggest the majority of encounters may be in the yard [9, 10]. However, yard-level densities of infected nymphs have not been predictive of tick-borne disease [4]. Interventions that reduced ticks in residential yards did not reduce tick encounters or disease, suggesting that people who experienced disease may have encountered ticks outside the yard, or in areas of the yard where pesticides were not applied [11].

Given the uncertainty about where people may encounter ticks, it is reasonable that public health officials provide general advice in their communications about how to reduce risk. The U.S. Centers for Disease Control, for example, have a web page on preventing ticks in the yard [13], while a separate page advises avoiding “wooded and brushy areas”, and walking “in the center of trails”, recommendations that could apply to any location [47]. Nevertheless, if some locations present higher risk, then personal protection or environmental interventions will likely be more effective when directed specifically at those high-risk areas. Interventions aimed at low-risk areas might not be as efficient or cost effective.

Our meta-analysis used objective and transparent procedures to identify 20 studies in eastern North America, spanning from 1981 to 2018, that identified the spatial scale of risk. In the absence of direct information in studies on the spatial scales of particular risk factors, we assigned variables to spatial scale where possible based on expectations about where activities typically take place [26, 27]. The meta-analysis revealed significant risk factors for exposure to infected blacklegged ticks at each of the three spatial scales: the yard (individual residential property), the neighborhood (area extending 500 m from the property boundary, not including the yard), and outside the neighborhood. Thus the analysis points to the need to attend to risk at each scale. However, risk was greatest at the neighborhood scale, followed by the yard and then outside the neighborhood.

One pathway by which factors in the neighborhood may influence risk is through human movements throughout the neighborhood, leading to exposure to ticks in neighbors’ yards and public areas beyond the yard. A second pathway by which neighborhood factors may influence risk is through movements by vertebrate hosts of ticks, as vertebrate hosts generally have home ranges much larger than an individual property in many residential settings [48–50]. Such movements could regularly import and export ticks between individual yards and the surrounding neighborhood, re-distributing risk through time. In both cases, interventions applied to individual yards would be less effective than those applied to both the yard and the surrounding neighborhood.

The movements of vertebrate hosts present a mechanism by which neighborhood-scale factors may influence yard-scale risk. Vertebrate hosts may thus contribute to neighborhood-scale factors being associated with higher odds ratios than yard-scale factors, yet people report acquiring most ticks in the yard [9, 10]. An alternate explanation for these patterns is that ticks may be encountered in the neighborhood at greater frequency than indicated by self-reports. The accuracy of self-reports about tick encounter location is constrained, in part, by people not immediately detecting ticks on them [51, 52].

Our results underscore the need to better characterize sources of variation in risk, through both the pathways of human mobility and vertebrate host communities, at the neighborhood scale [53]. The meta-analysis yielded six measures of risk at the neighborhood scale, compared to 104 measures at the other two spatial scales, indicating that risk at the neighborhood spatial scale is relatively under-studied. Presence of woods adjacent to or otherwise within 500 m of the yard represented four of six measures at the neighborhood scale, whereas there was a greater diversity of factors measured at the scales of the yard and outside the neighborhood. In the tick bite meta-analysis, two of three observations at the outside neighborhood scale pertained to use of permethrin-treated clothing, whereas there was a more varied set of factors at the yard scale (Fig. 4). While it is possible that differences between scales in the types of measures reported may have affected the spatial patterns found, we have no reason to expect a systematic bias in the literature toward more frequent selection of riskier variables at the neighborhood scale. Although the residential yard is physically situated within a neighborhood, which is found within a larger region, our analysis explicitly avoids nesting these three scales in order to identify the scales at which interventions are most likely to be effective. A neighborhood-scale intervention could involve a common action or set of actions being conducted in the respective yards of multiple residents in the neighborhood, as well as public spaces such as schools or parks.

Neighborhood definitions are commonly defined as the area within 500 m from home, this distance being based on walking accessibility [24, 54, 55]. Five hundred meters encompasses average walking distances to common destinations such as bus stops, supermarkets, or cemeteries [24]. We examined the effects of applying alternate neighborhood definitions using the available data from our meta-analysis. One study [56] presented data on risk associated with presence of woods at varying distances from property boundaries. We redid the analyses having categorized data from this study assuming either a neighborhood definition of 250 m or of 750 m. The results from these analyses did not substantively differ from those reached applying a neighborhood definition based on 500 m (Additional file 8). Data on time spent and tick encounters in the yard and at varying distances from home would enable future analyses on the areas where people are most at risk.

We found a modest decline in risk over time. We did not discern trends in study methodology that would explain this pattern. One potential cause of this pattern could be improvements in testing technology reducing the rate of false-positive diagnoses [57]. Alternatively, a recent decrease or stabilization in cases of Lyme disease reported to the U.S. Centers for Disease Control in states with high incidence may be due to local health departments reducing the resources allocated to verifying Lyme disease cases [2]. Another possibility is that over time, people have become more aware of how to avoid contacts with ticks.

A cause for caution in interpreting the results of the meta-analysis is the difficulty in inferring cause versus effect with respect to risk factors in retrospective studies, which look backwards to examine exposures to factors expected to increase or decrease risk. In prospective studies, in which cohorts are followed who differ in certain factors (e.g., a controlled trial of acaricide use in yards [11]), this problem is reduced. In our meta-analysis, 5 of 20 included studies were prospective [11, 18, 21, 58, 59]; the remaining studies were retrospective.

Our meta-analysis included studies from across eastern North America, but we did not incorporate potential regional differences in the model due to inadequate sample size across regions. It is possible that regional variation in Lyme disease incidence or ecology may have contributed to variation in results across studies. We note a possible pattern of relatively increased attention to yard-scale risk factors in locations in the highest quartile of Lyme disease incidence, and increased frequency of observations of factors outside the neighborhood in locations with Lyme disease incidence in the second quartile (Additional file 7: Table S5). However, the Chi-square test detected no relationship between the frequency of observations at each spatial scale and Lyme disease incidence. Most available studies were in the Northeast and Mid-Atlantic U.S., with one study in the Midwest U.S. [60] and one study in Canada [38].

Research to improve the diagnosis and treatment of Lyme disease and other tick-borne diseases is advancing rapidly, but fewer resources are allocated to avoidance and prevention of exposure. There were nearly five times as many articles on diagnosis and treatment of Lyme disease, anaplasmosis, and babesiosis as there were articles on prevention over the period 2005-2015 [61]. Efforts at prevention require knowledge of the spatial components of risk, specifically the locations used by both infected ticks and humans. Our meta-analysis suggests that risk-reducing interventions applied to any of the three spatial scales could be effective, but that those applied to the neighborhood scale are most likely to protect human health.

## Conclusions

Lyme disease and other tick-borne diseases are expanding their ranges in North America and elsewhere. Enhancing prevention requires understanding the spatial components of risk, including where and how infected ticks and humans come into contact. Our results highlight the need to better characterize sources of variation in risk at the neighborhood scale. We find that risk factors at the neighborhood scale are relatively under-studied, as are risk factors for tick bites at all scales. The meta-analyses suggest that risk-reducing interventions applied at each scale could be effective, but interventions applied at the neighborhood scale are most likely to protect human health. Interventions applied to individual yards would be less effective than those applied to both the yard and the surrounding neighborhood.

## Supplementary information

**Additional file 1.** PRISMA 2009 Checklist. Checklist for Preferred Reporting Items for Systematic Reviews and Meta-Analyses (PRISMA).

**Additional file 2: Table S1.** Possible risk factors found in initial set of six articles. “Variable” indicates risk factor as described in the article. We used these terms as keywords in a Web of Science search.

**Additional file 3.** Search strategy for identifying articles on spatial risk factors for people acquiring pathogenic *Ixodes scapularis*.

**Additional file 4: Table S2.** Spatial scale of a sample of incidence studies. For 50% of abstracts (619 randomly selected among the total 1237), we carried out full-text eligibility assessment for the 20 abstracts relating spatial risk factors to disease incidence. We determined the spatial scale of analysis for each study. (PDF 39 kb)

**Additional file 5: Table S3.** Estimated mean values, log values, and confidence intervals for each specific variable across all studies and spatial categories, including variables associated with both disease and tick bites. The table indicates those variables for which the 95% confidence intervals exclude one.

**Additional file 6: Table S4.** Overview of studies included in the meta-analysis of spatial risk factors for tick-borne diseases and tick bites.

**Additional file 7: Table S5.** Counts of measures at each spatial scale, relative to quartiles of Lyme disease incidence. Each count is the number of observations of disease risk factors at the specified spatial scale, and for studies in areas with Lyme disease incidence less than the quartile upper bound and greater than or equal to the upper bound of the previous quartile. Quartiles were computed based on the distribution of Lyme disease incidence in U.S. states (averaged over the years 2006-2016) and Canada (2009-2016).

**Additional file 8: Table S6.** Estimated odds ratio, standard error, 95% confidence interval, and *P*-values for effects of spatial scale and publication year on tick-borne disease risk, assuming neighborhood extends 750 m from property boundaries (**Table S6.1**) or 250 m from property boundaries (**Table S6.2**).

## Data Availability

The datasets analysed during the current study are available in the figshare repository: https://figshare.com/articles/Data_for_Systematic_review_and_meta-analysis_of_tick-borne_disease_risk_factors_in_residential_yards_neighborhoods_and_beyond/6081272.

## Abbreviations

AICc: Akaike Information Criterion for small samples
CI: Confidence intervals
PRISMA: Preferred Reporting Items for Systematic Reviews and Meta-Analyses
SE: Standard error

## References

[1] Nelson CA, Saha S, Kugeler KJ, Delorey MJ, Shankar MB, Hinckley AF (2015). Incidence of clinician-diagnosed Lyme disease, United States, 2005-2010. Emerg Infect Dis.

[2] Schwartz AS, Hinckley AF, Mead PS, Hook SA, Kugeler KJ (2017). Surveillance for Lyme disease - United States, 2008-2015. Morb Mortal Wkly Rep.

[3] Kugeler KJ, Farley GM, Forrester JD, Mead PS (2015). Geographic distribution and expansion of human Lyme disease, United States. Emerg Infect Dis.

[4] Connally NP, Ginsberg HS, Mather TN (2006). Assessing peridomestic entomological factors as predictors for Lyme disease. J Vector Ecol.

[5] Dister SW, Fish D, Bros SM, Frank DH, Wood BL (1997). Landscape characterization of peridomestic risk for Lyme disease using satellite imagery. Am J Trop Med Hyg.

[6] Cromley EK, Cartter ML, Mrozinski RD, Ertel S-H (1998). Residential setting as a risk factor for Lyme disease in a hyperendemic region. Am J Epidemiol.

[7] Eisen L, Dolan MC (2016). Evidence for personal protective measures to reduce human contact with blacklegged ticks and for environmentally based control methods to suppress host-seeking blacklegged ticks and reduce infection with Lyme disease spirochetes in tick vectors and rodent reservoirs. J Med Entomol.

[8] Ostfeld RS, Price A, Hornbostel VL, Benjamin MA, Keesing F (2006). Controlling ticks and tick-borne zoonoses with biological and chemical agents. Bioscience.

[9] Falco RC, Fish D (1988). Ticks parasitizing humans in a Lyme disease endemic area of southern New York state. Am J Epidemiol.

[10] Stafford KC, Williams SC, Molaei G (2017). Integrated pest management in controlling ticks and tick-associated diseases. J Integrated Pest Manag.

[11] Hinckley AF, Meek JI, Ray JA, Niesobecki SA, Connally NP, Feldman KA (2016). Effectiveness of residential acaricides to prevent Lyme and other tick-borne diseases in humans. J Infect Dis.

[12] Government of Canada (2015). Prevention of Lyme disease.

[13] Centers for Disease Control (2018). Preventing ticks in the yard.

[14] Stafford KC. Tick management handbook: an integrated guide for homeowners, pest control operators, and public health officials for the prevention of tick-associated disease. New Haven: Connecticut Agricultural Experiment Station; 2004.

[15] Gould LH, Nelson RS, Griffith KS, Hayes EB, Piesman J, Mead PS (2008). Knowledge, attitudes, and behaviors regarding Lyme disease prevention among Connecticut residents, 1999-2004. Vector Borne Zoonotic Dis.

[16] Garnett JM, Connally NP, Stafford KC, Cartter ML (2011). Student column: evaluation of deer-targeted interventions on Lyme disease incidence in Connecticut. Public Health Rep.

[17] Connally NP, Durante AJ, Yousey-Hindes KM, Meek JI, Nelson RS, Heimer R (2009). Peridomestic Lyme disease prevention: results of a population-based case-control study. Am J Prev Med.

[18] Finch C, Al-Damluji MS, Krause PJ, Niccolai L, Steeves T, O'Keefe CF (2014). Integrated assessment of behavioral and environmental risk factors for Lyme disease infection on Block Island. Rhode Island PLoS One.

[19] Klein JD, Eppes SC, Hunt P (1996). Environmental and life-style risk factors for Lyme disease in children. Clin Pediatr.

[20] Orloski KA, Campbell GL, Genese CA, Beckley JW, Schriefer ME, Spitalny KC (1998). Emergence of Lyme disease in Hunterdon County, New Jersey, 1993: a case-control study of risk factors and evaluation of reporting patterns. Am J Epidemiol.

[21] Smith G, Wileyto EP, Hopkins RB, Cherry BR, Maher JP (2001). Risk factors for Lyme disease in Chester County, Pennsylvania. Public Health Rep.

[22] Centers for Disease Control (2017). Lyme disease data tables: Reported cases of Lyme disease by state or locality, 2006-2016.

[23] Government of Canada. National Lyme disease surveillance in Canada 2013: web report 2015 [Available from: https://www.canada.ca/en/public-health/services/publications/diseases-conditions/national-lyme-disease-surveillance-canada-2013-web-report.html. Accessed 7 Oct 2019

[24] Hasanzadeh K, Broberg A, Kyttä M (2017). Where is my neighborhood? A dynamic individual-based definition of home ranges and implementation of multiple evaluation criteria. Appl Geogr.

[25] Salbach NM, O'Brien K, Brooks D, Irvin E, Martino R, Takhar P, Chan S, Howe JA (2014). Speed and Distance Requirements for Community Ambulation: A Systematic Review. Archives of Physical Medicine and Rehabilitation.

[26] Department of Interior (1991). 1991 National survey of fishing, hunting, and wildlife-associated recreation.

[27] Sonter Laura J., Watson Keri B., Wood Spencer A., Ricketts Taylor H. (2016). Spatial and Temporal Dynamics and Value of Nature-Based Recreation, Estimated via Social Media. PLOS ONE.

[28] Chaput EK, Meek JI, Heimer R (2002). Spatial analysis of human granulocytic ehrlichiosis near Lyme, Connecticut. Emerg Infect Dis.

[29] Frank DH, Fish D, Moy FH (1998). Landscape features associated with Lyme disease risk in a suburban residential environment. Landsc Ecol.

[30] Walsh MG (2013). The relevance of forest fragmentation on the incidence of human babesiosis: investigating the landscape epidemiology of an emerging tick-borne disease. Vector-Borne Zoonotic Dis.

[31] Jackson LE, Hilborn ED, Thomas JC (2006). Towards landscape design guidelines for reducing Lyme disease risk. Int J Epidemiol.

[32] Schwartz BS, Nadelman RB, Fish D, Childs JE, Forseter G, Wormser GP (1993). Entomologic and demographic correlates of antitick saliva antibody in a prospective study of tick bite subjects in Westchester County, New York. Am J Trop Med Hyg.

[33] Viechtbauer W (2010). Conducting meta-analyses in R with the metafor package. J Stat Softw.

[34] R Core Team (2018). R: A language and environment for statistical computing.

[35] Bland JM, Altman DG (2000). The odds ratio. Br Med J.

[36] Schwartz BS, Goldstein MD (1990). Lyme disease in outdoor workers: risk factors, preventive measures, and tick removal methods. Am J Epidemiol.

[37] Borenstein M, Hedges LV, Higgins JPT, Rothstein HR. Converting among effect sizes. In: Introduction to meta-analysis. Hoboken: Wiley; 2009. p. 45–9.

[38] Aenishaenslin C, Bouchard C, Koffi JK, Ogden NH (2017). Exposure and preventive behaviours toward ticks and Lyme disease in Canada: results from a first national survey. Ticks Tick Borne Diseases.

[39] Government of Canada (2018). Surveillance of Lyme disease.

[40] Anderson DR, Burnham KP (2002). Avoiding pitfalls when using information-theoretic methods. J Wildl Manag.

[41] Mazerolle MJ (2017). AICcmodavg: model selection and multimodel inference based on (Q)AIC(c).

[42] Koricheva J, Gurevitch J, Mengersen K. Handbook of meta-analysis in ecology and evolution. Princeton: Princeton University press; 2013.

[43] Green SB (1991). How many subjects does it take to do a regression analysis. Multivar Behav Res.

[44] Fischhoff IR, Bowden IR, Keesing F, Ostfeld RS. Data for: Systematic review and meta-analysis of tick-borne disease risk factors in residential yards, neighborhoods, and beyond 2018 [Available from: https://figshare.com/articles/Data_for_Systematic_review_and_meta-analysis_of_tick-borne_disease_risk_factors_in_residential_yards_neighborhoods_and_beyond/6081272]. 10.1186/s12879-019-4484-3 PMC6798452 31623574

[45] Mead P, Hook S, Niesobecki S, Ray J, Meek J, Delorey M (2018). Risk factors for tick exposure in suburban settings in the northeastern United States. Ticks Tick-Borne Dis.

[46] Eisen Lars, Eisen Rebecca J. (2016). Critical Evaluation of the Linkage Between Tick-Based Risk Measures and the Occurrence of Lyme Disease Cases: Table 1. Journal of Medical Entomology.

[47] Centers for Disease Control (2018). Preventing tick bites.

[48] Gross J, Elvinger F, Hungerford LL, Gehrt SD (2012). Raccoon use of the urban matrix in the Baltimore metropolitan area, Maryland. Urban Ecosyst.

[49] Kilpatrick HJ, Labonte AM, Barclay JS (2011). Effects of landscape and land-ownership patterns on deer movements in a suburban community. Wildl Soc Bull.

[50] Wright JD, Burt MS, Jackson VL (2012). Influences of an urban environment on home range and body mass of Virginia opossums (Didelphis virginiana). Northeast Nat.

[51] Rand PW, Lacombe EH, Dearborn R, Cahill B, Elias S, Lubelczyk CB (2007). Passive surveillance in Maine, an area emergent for tick-borne diseases. J Med Entomol.

[52] Falco RC, Fish D, Piesman J (1996). Duration of tick bites in a Lyme disease-endemic area. Am J Epidemiol.

[53] Keesing F, Ostfeld RS (2018). The tick project: testing environmental methods of preventing tick-borne diseases. Trends Parasitol.

[54] Larsen K, Gilliland J, Hess P, Tucker P, Irwin J, He M (2009). The influence of the physical environment and sociodemographic characteristics on children's mode of travel to and from school. Am J Public Health.

[55] Jago R, Baranowski T, Zakeri I, Harris M (2005). Observed environmental features and the physical activity of adolescent males. Am J Prev Med.

[56] Glass G E, Schwartz B S, Morgan J M, Johnson D T, Noy P M, Israel E (1995). Environmental risk factors for Lyme disease identified with geographic information systems. American Journal of Public Health.

[57] Hinckley AF, Connally NP, Meek JI, Johnson BJ, Kemperman MM, Feldman KA (2014). Lyme disease testing by large commercial laboratories in the United States. Clin Infect Dis.

[58] Richards SL, Balanay JAG, Harris JW (2015). Effectiveness of permethrin-treated clothing to prevent tick exposure in foresters in the central Appalachian region of the USA. Int J Environ Health Res.

[59] Vaughn MF, Meshnick SR (2011). Pilot study assessing the effectiveness of long-lasting permethrin-impregnated clothing for the prevention of tick bites. Vector-Borne Zoonotic Dis.

[60] Belongia E. A., Reed K. D., Mitchell P. D., Mueller-Rizner N., Vandermause M., Finkel M. F., Kazmierczak J. J. (2001). Tickborne Infections as a Cause of Nonspecific Febrile Illness in Wisconsin. Clinical Infectious Diseases.

[61] Sanchez E, Vannier E, Wormser GP, Hu LT (2016). Diagnosis, treatment, and prevention of Lyme disease, human granulocytic anaplasmosis, and babesiosis: a review. JAMA.

