# Correction to: Systematic review and meta-analysis of tick-borne disease risk factors in residential yards, neighborhoods, and beyond

**DOI:** 10.1186/s12879-019-4663-2

**Published:** 2019-12-05

**Authors:** Ilya R. Fischhoff, Sarah E. Bowden, Felicia Keesing, Richard S. Ostfeld

**Affiliations:** 1 Cary Institute of Ecosystem Studies, 2801 Sharon Turnpike, Millbrook, NY 12545 USA; 2 Eagle Medical Services, LLC, 2835 Brandywine Rd. Suite 200, Atlanta, GA 30341 USA; 3 Bard College, PO Box 5000, Annandale-on-Hudson, New York, 12504 USA

**Correction to: BMC Infect Dis (2019)19:861**

**https://doi.org/10.1186/s12879-019-4484-3**

Following publication of the original article [1], one of the authors, Dr. Sarah E. Bowden reported that at the time of the study she wasn’t working for the Division of Global Migration and Quarantine, Centers for Disease Control, 1600 Clifton Road, Atlanta, GA 30329–4027, USA. The institution should be removed from the Author details section.
