# Supplementary material for: Systematic review and meta-analysis of tick-borne disease risk factors in residential yards, neighborhoods, and beyond
Source: BMC Infect Dis. 2019 Oct 17;19:861. doi: 10.1186/s12879-019-4484-3 (PMC6798452; doi:10.1186/s12879-019-4484-3)
Supplement: Supplementary file 2 — Additional file 2: Table S1. Possible risk factors found in initial set of six articles. “Variable” indicates risk factor as described in the article. We used these terms as keywords in a Web of Science search. [file 12879_2019_4484_MOESM2_ESM.pdf]

Appendix Table A1. Possible risk factors found in initial set of six articles. "Variable" indicates risk factor as described in the article. We used these terms as keywords in a Web of Science search.

Variable	Source	keyword for Web of Science search
Groundhogs in yard	Klein et al. 1996	yard* groundhog*
black-legged ticks on property	Klein et al. 1996	propert*
leaf litter	Klein et al. 1996	leaf litter
Ground cover including moist humus	Klein et al. 1996	ground cover*
deer on property	Orloski et al. 1993	propert*
Deer damage landscape	Orloski et al. 1993	landscap*
woods	Connally et al. 2009	wood* OR propert* OR yard*
rock wall / stone wall	Smith et al. 2001	rock wall* OR stone wall*
Shrub percentage of land	Finch et al. 2014	shrub* OR land*
a yard or land attached to home	Smith et al. 2001	home* OR yard*
Deer damage to trees	Smith et al. 2001	tree* deer
Deer damage to shrubs	Smith et al. 2001	shrub* deer
Single family home	Smith et al. 2001	family OR home*
Rural residence	Orloski et al. 1993	rural* OR residen*
Suburban residence	Smith et al. 2001	suburb* OR residen*
Had woods adjacent to property	Connally et al. 2009	wood*
density of infected nymphs	Connally et al. 2006	density infected nymphs
age of home	Smith et al. 2001	home*
Age of nearby housing	Smith et al. 2001	hous*
Proximity to woods	Smith et al. 2001	wood*
mice observed	Smith et al. 2001	mice* observ*
bird feeder	Orloski et al. 1993	bird* feed*
presence of log pile / woodpile	Orloski et al. 1993	log* pile* OR woodpile*
presence of garden	Orloski et al. 1993	garden*
Landscape-related tick control measures	Finch et al. 2014	tick* control* OR landscap*
Clearing brush	Orloski et al. 1993	brush* OR clear*
Area-wide acaricide use	Finch et al. 2014	acaricide* use*
Wore light-colored clothing while in the yard	Smith et al. 2001	cloth*
Wore long pants while in the yard	Smith et al. 2001	long pant*
mulch or gravel dry barrier where lawn met woods	Connally et al. 2009	barrier* OR mulch* OR gravel* OR lawn*
Used pesticides for pests other than ticks	Connally et al. 2009	pesticide* use*
Trimmed branches back where lawn met woods	Connally et al. 2009	branch* OR trim*
Cleared leaf litter from where lawn met woods	Connally et al. 2009	leaf litter
Used rodent-targeted tick-control devices	Connally et al. 2009	tick* control*
Performed tick checks within 36 hours after spending time in the yard	Connally et al. 2009	tick* check*
Bathed within 2 hours after spending time in the yard	Connally et al. 2009	bath*
Wore repellent while in the yard	Connally et al. 2009	repellen* wore
Had deer-exclusion fencing around property	Connally et al. 2009	fenc*
Mowed lawn 3 or more times in the month prior to erythema migrans rash onset	Connally et al. 2009	mow* OR lawn*
Had a fence on the property (any type)	Connally et al. 2009	fenc*
Years in home	Smith et al. 2001	year* home*
Leave feed for mammals	Smith et al. 2001	feed* mammal*
Indices of gardening activity: use one or more electrical tools	Smith et al. 2001	garden*
Indices of gardening activity: use one or more gasoline tools	Smith et al. 2001	garden*
Unpowered tools	Smith et al. 2001	unpowered tool*
Hour per week yard work	Smith et al. 2001	work* OR yard*
before working or playing in yard: apply insect repellents	Smith et al. 2001	play*
before working or playing in yard: wear long pants	Smith et al. 2001	long pant*
before working or playing in yard: wear light-colored clothing	Smith et al. 2001	light* cloth*
before working or playing in yard: use acaricides	Smith et al. 2001	acaricide* use*
Occupational exposure to tick habitat	Finch et al. 2014	occupat* OR expos*
Have outdoor job	Smith et al. 2001	outdoor* OR job*
Have a job that takes you into the woods	Smith et al. 2001	job* OR wood*

Appendix Table A1. Possible risk factors found in initial set of six articles. "Variable" indicates risk factor as described in the article. We used these terms as keywords in a Web of Science search.

Variable	Source	keyword for Web of Science search
Have a job that takes you into woody margins	Smith et al. 2001	wood* OR margin*
Visit parks	Smith et al. 2001	park* OR visit*
Camp in an RV	Smith et al. 2001	camp*
Picnic in designated areas in parks	Smith et al. 2001	picnic*
before work or recreation outdoors away from home: apply insect repellents	Smith et al. 2001	recreat*
before work or recreation outdoors away from home: wear long pants	Smith et al. 2001	long pant*
before work or recreation outdoors away from home: wear light colored clothing	Smith et al. 2001	light* cloth*
before work or recreation outdoors away from home: tuck pants legs into socks	Smith et al. 2001	sock* OR pant*
before work or recreation outdoors away from home: use acaricides	Smith et al. 2001	acaricide* use*
Work in wooded areas, hours per week	Smith et al. 2001	work* wood*
average age of people with cases	Finch et al. 2014	age*
Female or Male	Orloski et al. 1993	female* OR male* OR gender*
hours spent in vegetation	Finch et al. 2014	hour* vegetat*
owning a dog	Finch et al. 2014	dog* own*
owning a cat	Finch et al. 2014	cat* own*
owning a horse	Finch et al. 2014	horse*
owning a different pet	Finch et al. 2014	pet* own*
previous Lyme diagnosis	Finch et al. 2014	Lyme diagnos* previous*
Use of any protective measure	Finch et al. 2014	protect* measure*
use of repellent	Finch et al. 2014	repellen* use*
use of protective clothing	Finch et al. 2014	protect* OR cloth*
wear long pants	Connally et al. 2009	long pant*
wear light-colored clothing	Connally et al. 2009	light cloth*
Tuck pants into socks	Connally et al. 2009	pant* OR socks
Avoiding brush	Finch et al. 2014	avoid* OR brush*
Tick checking	Orloski et al. 1993	tick* check*
Tick bite within the past year	Finch et al. 2014	tick* bite*
Tick bite within the past year in study area	Finch et al. 2014	tick* bite*
Received prophylactic antibiotic therapy after tick bite	Connally et al. 2009	tick* bite*
camping	Klein et al. 1996	camp*
Does using woods in or near the yard for recreation or play increase the risk?	Smith et al. 2001	wood*
Being a parent	Smith et al. 2001	parent*
Have children who participate in outdoor sports	Smith et al. 2001	outdoor*
Engage in any outdoor recreation	Smith et al. 2001	outdoor* OR recreat*
Fish	Klein et al. 1996	fish*
Hunt	Smith et al. 2001	hunt*
Ride Horses	Smith et al. 2001	horse*
Camp in a tent	Smith et al. 2001	tent*
Walk or jog outdoors	Smith et al. 2001	jog* OR walk*
Walk or jog in woods or wooded areas	Orloski et al. 1993	walk* OR wood* OR jog* OR wood*
walk or jog in grassy areas	Orloski et al. 1993	walk* OR grass* OR jog* OR grass*
play on mowed fields	Orloski et al. 1993	play* OR field* OR play* OR mow*
Picnic outside parks or in non-designated areas in parks	Smith et al. 2001	picnic*
check for ticks during outdoor activites	Smith et al. 2001	tick* check* OR outdoor* OR activit*
check for ticks after outdoor activities	Smith et al. 2001	tick* check* OR outdoor* OR activit*
finding ticks during a search	Smith et al. 2001	outdoor*
trimmed branches back where lawn met woods	Connally et al. 2009	trim* OR branch*
cleared leaf litter where lawn met woods	Connally et al. 2009	clear* OR leaf litter
time outdoors in warm weather	Klein et al. 1996	outdoor*
vacationing in Lyme-endemic regions	Klein et al. 1996	vacation*

Appendix Table A1. Possible risk factors found in initial set of six articles. "Variable" indicates risk factor as described in the article. We used these terms as keywords in a Web of Science search.

Variable	Source	keyword for Web of Science search
school attendance	Klein et al. 1996	school*
daycare attendance	Klein et al. 1996	daycare*
hiking or playing in woods	Klein et al. 1996	hik* OR play*
rolling down hills	Klein et al. 1996	roll* OR hill*
climbing trees	Klein et al. 1996	climb* OR tree*
playing in yard	Klein et al. 1996	play*
running or jogging cross-country	Klein et al. 1996	run* OR jog*
public playground use	Klein et al. 1996	public* OR play*
participation in outdoor sports	Klein et al. 1996	sport* OR outdoor*
golf	Klein et al. 1996	golf*
summer camp attendance	Klein et al. 1996	camp*
lawn mowing	Klein et al. 1996	lawn* OR mow*
wood cutting	Klein et al. 1996	cut* OR wood*
leaf raking	Klein et al. 1996	rak*
gardening	Klein et al. 1996	garden*
burning vegetation	Klein et al. 1996	vegetat* OR burn*
parent with outdoor occupation	Klein et al. 1996	parent*
presence on property of animals (including raccoons, opossums, birds, mice, deer)	Klein et al. 1996	raccoon* presen* OR opossum* presen* OR bird* presen*
owning pets	Klein et al. 1996	pet* own*
identification of deer ticks on pets	Klein et al. 1996	tick* pet*
identification of deer ticks on children	Klein et al. 1996	tick* child* OR child*
other insect bites	Klein et al. 1996	insect* bite*
use of insect repellents on children	Klein et al. 1996	repellen* use*
use of insecticides on property	Klein et al. 1996	insecticid* use*
use of protective dress (long pants, shoes, and socks in warm weather)	Klein et al. 1996	protect* use*
regular tick checks	Klein et al. 1996	tick* check*
frequency of bathing (including by parent)	Klein et al. 1996	bath*
description of child as extrovert, outdoor type, nature lover, or animal lover	Klein et al. 1996	natur*
recreational areas (e.g. playscapes)	Connally et al. 2009	recreat*
wear clothing treated with permethrin insecticide	Connally et al. 2009	permethrin
history of travel to tick-endemic areas	Connally et al. 2009	travel*
deer ticks on property	Klein et al. 1996	propert* tick*
own livestock	Connally et al. 2009	livestock* own*
entomologic risk index	Connally et al. 2006	entomologic* risk*
