# Supplementary material for: Systematic review and meta-analysis of tick-borne disease risk factors in residential yards, neighborhoods, and beyond
Source: BMC Infect Dis. 2019 Oct 17;19:861. doi: 10.1186/s12879-019-4484-3 (PMC6798452; doi:10.1186/s12879-019-4484-3)
Supplement: Supplementary file 3 — Additional file 3. Search strategy for identifying articles on spatial risk factors for people acquiring pathogenic Ixodes scapularis. [file 12879_2019_4484_MOESM3_ESM.pdf]

Appendix S3. Search strategy for identifying articles on spatial risk factors for people acquiring pathogenic *Ixodes scapularis*

<u>Criteria</u>:

- Location:
  - U.S. states where Lyme incidence was at least 1 per 100,000 in 2015, the most recently reported year [1]
  - Canadian provinces where incidence was at least 1 per 100,000 in 2013, the most recently reported year [2]
- Risk factors:
  - Keywords identified from initial set of six articles
  - Additional keywords identified by authors to broaden search
  - Exclude: Articles on irrelevant locations, hosts, vectors, or on topics (e.g., physiology, symptoms, treatment)
- Vector / disease: *Ixodes scapularis* or pathogens transmitted by *I. scapularis*
- Language: English
- Publication type: Article or Book Chapter or Data paper

<u>Advanced search in Web of Science matching above criteria</u>:

TS = ((United States OR Pennsylvania OR Connecticut OR Delaware OR District of Columbia OR Illinois OR Iowa OR Indiana OR Maine OR Maryland OR Massachusetts OR Michigan OR Minnesota OR New Hampshire OR New Jersey OR New York OR North Dakota OR Ohio OR Rhode Island OR Vermont OR Virginia OR Wisconsin OR
Canad* OR Manitoba OR Ontario OR Quebec OR Nova Scotia OR
risk* factor*)
AND
(*Ixodes scapularis* OR *Ixodes dammini* OR Lyme OR Borreli* OR Babesi* OR Powassa* OR Anaplasm*) AND
(risk* factor* OR case-control OR Odds Ratio OR relative risk OR peridomestic* OR nonperidomestic* OR environment* OR behav* OR demograph* OR life-style OR lifestyle OR Tick avoid* OR prevent* OR expos* OR neighbor* OR hik* OR

yard* groundhog* OR
propert* OR
leaf litter OR
ground cover* OR
propert* OR
landscap* OR

wood* OR propert* OR yard* OR
rock wall* OR stone wall* OR
shrub* OR land* OR
home* OR yard* OR
tree* deer OR
shrub* deer OR
family OR home* OR
rural* OR residen* OR
suburb* OR residen* OR
wood* OR
density infected nymphs OR
home* OR
hous* OR
wood* OR
mice* observ* OR
bird* feed* OR
log* pile* OR woodpile* OR
garden* OR
tick* control* OR landscap* OR
brush* OR clear* OR
acaricide* use* OR
cloth* OR
long pant* OR
barrier* OR mulch* OR gravel* OR lawn* OR
pesticide* use* OR
branch* OR trim* OR
leaf litter OR
tick* control* OR
tick* check* OR
bath* OR
repellen* wore OR
fenc* OR

mow* OR lawn* OR
fenc* OR
year* home* OR
feed* mammal* OR
garden* OR
garden* OR
unpowered tool* OR
work* OR yard* OR
play* OR
long pant* OR
light* cloth* OR
acaricide* use* OR
occupat* OR expos* OR
outdoor* OR job* OR
job* OR wood* OR
wood* OR margin* OR
park* OR visit* OR
camp* OR
picnic* OR
recreat* OR
long pant* OR
light* cloth* OR
sock* OR pant* OR
acaricide* use* OR
work* wood* OR
age* OR
race* OR
female* OR male* OR gender* OR
hour* spent vegetation
dog* own* OR
cat* own* OR
horse* OR

pet* own* OR
Lyme diagnos* previous* OR
protect* measure* OR
repellen* use* OR
protect* OR cloth* OR
long pant* OR
light cloth* OR
pant* OR socks OR
avoid* OR brush* OR
tick* check* OR
tick* bite* OR
tick* bite* OR
tick* bite* OR
camp* OR
wood* OR
parent* OR
outdoor* OR
outdoor* OR recreat* OR
fish* OR
hunt* OR
horse* OR
tent* OR
jog* OR walk* OR
walk* OR wood* OR jog* OR wood* OR
walk* OR grass* OR jog* OR grass* OR
play* OR field* OR play* OR mow* OR
hik* OR
picnic* OR
tick* check* OR outdoor* OR activit* OR
tick* check* OR outdoor* OR activit* OR
outdoor* OR
trim* OR branch* OR

clear* OR leaf litter OR
pesticide* OR
outdoor* OR
vacation* OR
school* OR
daycare* OR
hik* OR play* OR
roll* OR hill* OR
climb* OR tree* OR
play* OR
run* OR jog* OR
public* OR play* OR
sport* OR outdoor* OR
golf* OR
camp* OR
lawn* OR mow* OR
cut* OR wood* OR
rak* OR
garden* OR
vegetat* OR burn* OR
parent* OR
raccoon* presen* OR opossum* presen* OR bird* presen* OR
pet* own* OR
tick* pet* OR
tick* child* OR child* OR
insect* bite* OR
repellen* use* OR
insecticid* use* OR
protect* use* OR
tick* check* OR
bath* OR

notif* physician* febril* OR
natur* OR
recreat* OR
permethrin OR
travel* OR
propert* tick* OR
livestock* own* OR
entomologic* risk* OR
Ethnic* OR
socioeconom*

)
NOT
(California OR Europe* OR Austria OR Italy OR Belgium OR Latvia OR Bulgaria OR Lithuania OR Croatia OR Luxembourg OR Cyprus OR Malta OR Czech Republic OR Netherlands OR Denmark OR Poland OR Estonia OR Portugal OR Finland OR Romania OR France OR Slovakia OR Germany OR Slovenia OR Greece OR Spain OR Hungary OR Sweden OR Ireland OR United Kingdom OR Switzerland OR China OR Africa* OR Kenya OR Brazil OR Turkey OR Zimbabwe OR India OR Ghana OR Sri Lanka OR Asia*))
NOT
TI = (canine OR dog* OR horse* OR cat* OR feline OR chicken* OR fox* OR raccoon* OR bird* OR
Rhipicephalus OR *Boophilus OR* Amblyomma OR Argasidae OR Dermacentor OR *Haemaphysalis* OR Ixodes pacificus OR affinus OR
host OR detection OR surg* OR blood* OR genom* OR enzym* OR RNA OR reservoir OR DNA OR microb* OR card* OR molec* OR protein*)

AND Language: English
AND publication type: Article OR Book Chapter OR Data Paper OR Proceedings Paper)

References cited

1. CDC. Lyme disease data tables: Reported cases of Lyme disease by state or locality, 2006-2016. https://www.cdc.gov/lyme/stats/tables.html (accessed 1 July 2017).
2. Canada. National Lyme Disease Surveillance in Canada 2013: Web Report. https://www.canada.ca/en/public-health/services/publications/diseases-conditions/national-lyme-disease-surveillance-canada-2013-web-report.html (accessed 1 July 2017).
