# Supplementary material for: Systematic review and meta-analysis of tick-borne disease risk factors in residential yards, neighborhoods, and beyond
Source: BMC Infect Dis. 2019 Oct 17;19:861. doi: 10.1186/s12879-019-4484-3 (PMC6798452; doi:10.1186/s12879-019-4484-3)
Supplement: Supplementary file 4 — Additional file 4: Table S2. Spatial scale of a sample of incidence studies. For 50% of abstracts (619 randomly selected among the total 1237), we carried out full-text eligibility assessment for the 20 abstracts relating spatial risk factors to disease incidence. We determined the spatial scale of analysis for each study. (PDF 39 kb) [file 12879_2019_4484_MOESM4_ESM.pdf]

Appendix Table A2. Spatial scale of a sample of incidence studies. For 50% of abstracts (619 randomly selected among the total 1,237), we carried out full-text eligibility assessment for the 20 abstracts relating spatial risk factors to disease incidence. We determined the spatial scale of analysis for each study.

citation	spatial scale for incidence data
L.E. Jackson, E.D. Hilborn, J.C. Thomas, Towards landscape design guidelines for reducing Lyme disease risk, Int. J. Epidemiol. 35 (2006) 315–322. doi:10.1093/ije/dyi284.	areas bounded by federally funded roads, 0.02 to 580 km2
E.K. Cromley, M.L. Cartter, R.D. Mrozinski, S.-H. Ertel, Residential Setting as a Risk Factor for Lyme Disease in a Hyperendemic Region, Am. J. Epidemiol. 147 (1998) 472–477.	inside vs. outside village
Nicholson, T. & Mather, M. (1996) Methods for evaluating Lyme disease risks using geographic information systems and geospatial analysis. Journal of Medical Entomology, 33, 711–719.	10 km2 quadrat
R.C. Falco, D.F. McKenna, T.J. Daniels, R.B. Nadelman, J. Nowakowski, D. Fish, G.P. Wormser, Temporal Relation between Ixodes scapularis Abundance and Risk for Lyme Disease Associated with Erythema Migrans, Am. J. Epidemiol. 149 (1999) 771–776. doi:10.1093/oxfordjournals.aje.a009886.	county
Greene SK, Levin-Rector A, Hadler JL, Fine AD. Disparities in Reportable Communicable Disease Incidence by Census Tract-Level Poverty, New York City, 2006-2013. Am J Public Health. 2015; 105(9):E27–E34.	census tract
Kilpatrick HJ, Labonte AM, Stafford III KC. The Relationship Between Deer Density, Tick Abundance, and Human Cases of Lyme Disease in a Residential Community. J Med Entomol. 2014; 51(4):777–784.	community
Seukep SE, Kolivras KN, Hong Y, et al. An Examination of the Demographic and Environmental Variables Correlated with Lyme Disease Emergence in Virginia. Ecohealth. 2015; 12(4):634–644.	census tract
Walsh MG. The Relevance of Forest Fragmentation on the Incidence of Human Babesiosis: Investigating the Landscape Epidemiology of an Emerging Tick-Borne Disease. Vector-borne zoonotic Dis. 2013; 13(4):250–255.	county
Dahlgren FS, Mandel EJ, Krebs JW, Massung RF, McQuiston JH. Increasing Incidence of Ehrlichia chaffeensis and Anaplasma phagocytophilum in the United States, 2000-2007. Am J Trop Med Hyg. 2011; 85(1):124–131.	state
Wiznia D, Christos P, LaBonte A. The Use of Deer Vehicle Accidents as a Proxy for Measuring the Degree of Interaction Between Human and Deer Populations and Its Correlation With the Incidence Rate of Lyme Disease. J Environ Health. 2014; 75(8):32–39.	town; deer management zone
Levi T, Kilpatrick AM, Mangel M, Wilmers CC. Deer, predators, and the emergence of Lyme disease. Proc Natl Acad Sci U S A. 2012; 109(27):10942–10947.	county

Appendix Table A2. Spatial scale of a sample of incidence studies. For 50% of abstracts (619 randomly selected among the total 1,237), we carried out full-text eligibility assessment for the 20 abstracts relating spatial risk factors to disease incidence. We determined the spatial scale of analysis for each study.	
citation	spatial scale for incidence data
Garnett JMJ, Connally NNP, Stafford K, Cartter MML, Stafford III KC, Cartter MML. Student Column: Evaluation of deer-targeted interventions on Lyme disease incidence in Connecticut. Public Health Rep. 2011; 126(3):446–454.	town; county
Hilborn ED, Catanzaro DG, Jackson LE. Repeated holdout cross-validation of model to estimate risk of Lyme disease by landscape characteristics. Int J Environ Health Res. 2012; 22(1):1–11.	federally funded roads, 0.02 to 580 km2
Schauber EM, Ostfeld RS, Evans AS. What is the best predictor of annual Lyme disease incidence: Weather, mice, or acorns? Ecol Appl. 2005; 15(2): 575–586.	state
Chaput EK, Meek JI, Heimer R. Spatial analysis of human granulocytic ehrlichiosis near Lyme, Connecticut. Emerg Infect Dis. 2002; 8(9):943–948.	census block group
Naleway, A. L., et al. (2002). "Lyme disease incidence in Wisconsin: A comparison of state-reported rates and rates from a population-based cohort." AMERICAN JOURNAL OF EPIDEMIOLOGY 155(12): 1120-1127.	town, county
Frank Mapping Lyme disease incidence for diagnostic and preventive decisions, Maryland	zip code
Stafford, K. C., et al. (1998). "Temporal correlations between tick abundance and prevalence of ticks infected with Borrelia burgdorferi and increasing incidence of Lyme disease." JOURNAL OF CLINICAL MICROBIOLOGY 36 (5): 1240-1244.	town
Kitron, U. and J. J. Kazmierczak (1997). "Spatial analysis of the distribution of Lyme disease in Wisconsin." AMERICAN JOURNAL OF EPIDEMIOLOGY 145(6): 558-566.	county
