# Supplementary material for: Systematic review and meta-analysis of tick-borne disease risk factors in residential yards, neighborhoods, and beyond
Source: BMC Infect Dis. 2019 Oct 17;19:861. doi: 10.1186/s12879-019-4484-3 (PMC6798452; doi:10.1186/s12879-019-4484-3)
Supplement: Supplementary file 5 — Additional file 5: Table S3. Estimated mean values, log values, and confidence intervals for each specific variable across all studies and spatial categories, including variables associated with both disease and tick bites. The table indicates those variables for which the 95% confidence intervals exclude one. [file 12879_2019_4484_MOESM5_ESM.pdf]

Appendix Table A3. Estimated mean values, log values, and confidence intervals for each specific variable across all studies and spatial categories, including variables associated with both disease and tick bites. The table indicates those variables for which the 95% confidence intervals exclude one.

variable	log.odds.ratio	log.standard.err	log.ci.ub	log.ci.lb	log.odds.negative	odds.ratio	se	ci.upper.bound	ci.lower.bound	odds ratio CI excludes 1
clear brush	1.25	0.56	2.35	0.14	0	3.49	1.751	10.486	1.15	1
deer damage to landscape	0.89	0.41	1.69	0.08	0	2.435	1.507	5.419	1.083	1
deer on property	1.25	0.33	1.91	0.6	0	3.49	1.391	6.753	1.822	1
hiking or camping	1.33	0.68	2.66	0.01	0	3.781	1.974	14.296	1.01	1
permethrin-treated clothing	-2.69	0.77	-1.18	-4.19	1	0.068	2.16	0.307	0.015	1
single family home	0.91	0.44	1.77	0.05	0	2.484	1.553	5.871	1.051	1
woodpile	0.84	0.33	1.49	0.19	0	2.316	1.391	4.437	1.209	1
woods	0.69	0.18	1.04	0.34	0	1.994	1.197	2.829	1.405	1
age nearby housing	0.98	0.53	2.01	-0.05	0	2.664	1.699	7.463	0.951	0
age of home	0.69	0.44	1.56	-0.18	0	1.994	1.553	4.759	0.835	0
bathed	-0.51	0.44	0.36	-1.38	1	0.6	1.553	1.433	0.252	0
before activity: use acaricides	0.33	0.31	0.94	-0.28	0	1.391	1.363	2.56	0.756	0
bird feeder	0.23	0.19	0.61	-0.14	0	1.259	1.209	1.84	0.869	0
blacklegged ticks on property	0.88	0.61	2.06	-0.31	0	2.411	1.84	7.846	0.733	0
camp in a tent	-0.4	0.43	0.45	-1.25	1	0.67	1.537	1.568	0.287	0
camp in an rv	-0.04	0.49	0.91	-1	1	0.961	1.632	2.484	0.368	0
camping	0.58	0.48	1.52	-0.36	0	1.786	1.616	4.572	0.698	0
clear litter where lawn met woods	0.32	0.41	1.13	-0.49	0	1.377	1.507	3.096	0.613	0
courtyard, garden or wooded	0.8	0.53	1.85	-0.24	0	2.226	1.699	6.36	0.787	0
deer damage landscape	0.71	0.55	1.79	-0.36	0	2.034	1.733	5.989	0.698	0
density of infected nymphs	0.11	0.38	0.85	-0.64	0	1.116	1.462	2.34	0.527	0
dry barrier: lawn met woods	-0.35	0.45	0.54	-1.24	1	0.705	1.568	1.716	0.289	0
fence (deer-exclusion)	-0.14	0.49	0.81	-1.09	1	0.869	1.632	2.248	0.336	0
fence on property (any)	-0.24	0.41	0.56	-1.03	1	0.787	1.507	1.751	0.357	0
fishing	0.3	0.32	0.92	-0.32	0	1.35	1.377	2.509	0.726	0
frequency of yard work	0.7	0.36	1.41	0	0	2.014	1.433	4.096	1	0
garden	0.19	0.27	0.72	-0.35	0	1.209	1.31	2.054	0.705	0
gardening	0.04	0.48	0.98	-0.9	0	1.041	1.616	2.664	0.407	0
gardening activity tools	0.97	0.51	1.97	-0.04	0	2.638	1.665	7.171	0.961	0
ground cover incl. moist humus	0.94	0.6	2.11	-0.23	0	2.56	1.822	8.248	0.795	0
groundhogs	-0.87	0.58	0.27	-2.01	1	0.419	1.786	1.31	0.134	0
hiking	0.68	0.48	1.62	-0.26	0	1.974	1.616	5.053	0.771	0
hours in someone else's yard	0.1	0.38	0.85	-0.66	0	1.105	1.462	2.34	0.517	0
hunting	0.29	0.28	0.84	-0.27	0	1.336	1.323	2.316	0.763	0
landscape tick control	-0.01	0.46	0.88	-0.91	1	0.99	1.584	2.411	0.403	0
leaf litter	1.06	0.63	2.29	-0.17	0	2.886	1.878	9.875	0.844	0
leave feed for animals	0.53	0.44	1.38	-0.32	0	1.699	1.553	3.975	0.726	0
light-colored clothing	-0.09	0.24	0.38	-0.57	1	0.914	1.271	1.462	0.566	0
log pile	0.12	0.4	0.91	-0.67	0	1.127	1.492	2.484	0.512	0
long pants	-0.07	0.23	0.37	-0.52	1	0.932	1.259	1.448	0.595	0
long-sleeved shirt	0.33	0.57	1.44	-0.78	0	1.391	1.768	4.221	0.458	0
mice observed	0.68	0.41	1.48	-0.13	0	1.974	1.507	4.393	0.878	0
mowed lawn	0.36	0.42	1.19	-0.47	0	1.433	1.522	3.287	0.625	0

Appendix Table A3. Estimated mean values, log values, and confidence intervals for each specific variable across all studies and spatial categories, including variables associated with both disease and tick bites. The table indicates those variables for which the 95% confidence intervals exclude one.

variable	log.odds.ratio	log.standard.er	log.ci.ub	log.ci.lb	log.odds.negative	odds.ratio	se	ci.upper.bound	ci.lower.bound	odds ratio CI excludes 1
**occupational tick exposure**	0.38	0.24	0.84	-0.08	0	1.462	1.271	2.316	0.923	0
**outdoor dining area**	0.79	0.51	1.78	-0.2	0	2.203	1.665	5.93	0.819	0
**outdoor work hours**	0.18	0.48	1.12	-0.76	0	1.197	1.616	3.065	0.468	0
**pesticides for non-ticks**	-0.16	0.41	0.65	-0.98	1	0.852	1.507	1.916	0.375	0
**picnic in parks**	0.16	0.41	0.95	-0.64	0	1.174	1.507	2.586	0.527	0
**picnic outside parks**	0.39	0.42	1.2	-0.43	0	1.477	1.522	3.32	0.651	0
**property size>1 but less than 2 acres**	0.1	0.51	1.1	-0.91	0	1.105	1.665	3.004	0.403	0
**repellent**	-0.26	0.23	0.19	-0.7	1	0.771	1.259	1.209	0.497	0
**ride horses**	0.21	0.48	1.15	-0.73	0	1.234	1.616	3.158	0.482	0
**rock wall**	0.33	0.23	0.78	-0.11	0	1.391	1.259	2.181	0.896	0
**rodent-targeted tick-control**	0.37	0.62	1.59	-0.85	0	1.448	1.859	4.904	0.427	0
**shrub percentage of land**	0.21	0.44	1.07	-0.66	0	1.234	1.553	2.915	0.517	0
**sprayed acaricide**	0.05	0.22	0.49	-0.39	0	1.051	1.246	1.632	0.677	0
**tick checking**	-0.32	0.34	0.35	-0.99	1	0.726	1.405	1.419	0.372	0
**time spent in yard**	0.37	0.33	1.02	-0.28	0	1.448	1.391	2.773	0.756	0
**trim branches: lawn met woods**	-0.15	0.44	0.72	-1.02	1	0.861	1.553	2.054	0.361	0
**tuck pants into socks**	0.12	0.85	1.79	-1.55	0	1.127	2.34	5.989	0.212	0
**tuck pants legs into socks**	0.09	0.31	0.7	-0.52	0	1.094	1.363	2.014	0.595	0
**visit parks**	-0.67	0.44	0.18	-1.53	1	0.512	1.553	1.197	0.217	0
**visited high risk region**	0.57	0.49	1.53	-0.38	0	1.768	1.632	4.618	0.684	0
**yard/land attached to home**	1.51	0.8	3.08	-0.06	0	4.527	2.226	21.758	0.942	0
