# Supplementary material for: Systematic review and meta-analysis of tick-borne disease risk factors in residential yards, neighborhoods, and beyond
Source: BMC Infect Dis. 2019 Oct 17;19:861. doi: 10.1186/s12879-019-4484-3 (PMC6798452; doi:10.1186/s12879-019-4484-3)
Supplement: Supplementary file 6 — Additional file 6: Table S4. Overview of studies included in the meta-analysis of spatial risk factors for tick-borne diseases and tick bites. [file 12879_2019_4484_MOESM6_ESM.pdf]

Appendix Table A4. Overview of studies included in the meta-analysis of spatial risk factors for tick-borne diseases and tick bites.

| Citation | Cases | Controls | Age | Study dates | Outcome measure | Study type | Location |
|---|---|---|---|---|---|---|---|
| Aenishaenslin et al. 2017 [1] | 42 | 2834 | All | 2014 | disease | survey | Canada |
| Connally et al. 2009 [2] | 364 | 349 | All | 2005-2007 | disease | case control | CT |
| Mead et al. 2018[3] | 91 | 840 | All | 2013 | bite | case control | CT |
| Vazquez et al. 2008 [4] | 285 | 557 | All | 2003 | disease | case control | CT |
| Hinckley et al. 2015 bite [5] | 1303 | 2502 | All | 2011-2012 | bite | controlled trial | CT, MD, NY |
| Hinckley et al. 2015 disease [5] | 1273 | 2461 | All | 2011-2012 | disease | controlled trial | CT, MD, NY |
| Klein et al. 1996 [6] | 42 | 42 | Children | not specified | disease | case control | DE, MD, PA, NJ |
| Richards et al. 2015 [7] | 22 | 13 | Adults | 2013 | bite | controlled trial | KY, NC, OH, TN, VA, WV |
| Glass et al. 1995 [8] | 47 | 492 | not specified | 1989-1990 | disease | case control | MD |
| Bowen et al. 1984 [9] | 20 | 1112 | Adults | 1981-1982 | disease | case control | NJ |
| Schwartz & Goldstein 1990 [10] | 39 | 633 | Adults | 1988 | disease | case control | NJ |
| Schwartz et al. 1991 [11] | 22 | 213 | Adults | 1989 | bite | case control | NJ |
| Orloski et al. 1998 [12] | 51 | 51 | All | 1993 | disease | case control | NJ |
| Wallace et al. 1998 [13] | 19 | 53 | Adults | 1995 | disease | case control | NY |
| Townsend et al. 2003 [14] | 88 | 492 | All | 2001-2002 | disease | case control | NY |
| Smith et al. 1988 [15] | 27 | 362 | Adults | 1986 | disease | longitudinal | NY |
| Vaughn & Mesnick 2011 [16] | 9 | 7 | Adults | 2008 | bite | non-controlled trial | NC |
| Smith et al. 2001 [17] | 294 | 449 | All | 1998 | disease | case control | PA |
| Connally et al. 2006 [18] | 47 | 47 | All | 2002-2003 | disease | case control | RI |
| Finch et al. 2014 [19] | 86 | 809 | not specified | 2005-2011 | disease | longitudinal | RI |
| Belongia et al. 2001 [20] | 17 | 45 | All | 1997-1998 | disease | case control | WI |

## References


1. Aenishaenslin, C., et al., *Exposure and preventive behaviours toward ticks and Lyme disease in Canada: Results from a first national survey.* Ticks Tick Borne Diseases, 2017. **8**(1): p. 112-118.
2. Connally, N.P., et al., *Peridomestic Lyme disease prevention: results of a population-based case-control study.* Am J Prev Med, 2009. **37**(3): p. 201-6.
3. Mead, P., et al., *Risk factors for tick exposure in suburban settings in the Northeastern United States.* Ticks and Tick-Borne Diseases, 2018. **9**(2): p. 319-324.
4. Vazquez, M., et al., *Effectiveness of Personal Protective Measures to Prevent Lyme Disease.* Emerging Infectious Diseases, 2008. **14**(2): p. 210-216.
5. Hinckley, A.F., et al., *Effectiveness of Residential Acaricides to Prevent Lyme and Other Tick-borne Diseases in Humans.* J Infect Dis, 2016. **214**(2): p. 182-8.
6. Klein, J.D., S.C. Eppes, and P. Hunt, *Environmental and Life-style Risk Factors for Lyme Disease in Children.* Clinical Pediatrics, 1996. **35**(7): p. 359-363.
7. Richards, S.L., J.A.G. Balanay, and J.W. Harris, *Effectiveness of permethrin-treated clothing to prevent tick exposure in foresters in the central Appalachian region of the USA.* International Journal of Environmental Health Research, 2015. **25**(4): p. 453-462.
8. Glass, G.E., et al., *Environmental Risk Factors for Lyme Disease Identified with Geographic Information Systems.* American Journal of Public Health, 1994.
9. Bowen, G.S., et al., *A Focus of Lyme diseases in Monmouth County, New Jersey.* American Journal of Epidemiology, 1984. **120**(3): p. 387-394.
10. Schwartz, B.S. and M.D. Goldstein, *Lyme disease in outdoor workers: risk factors, preventive measures, and tick removal methods.* American Journal of Epidemiology, 1990. **131**(5): p. 877-885.
11. Schwartz, B.S., et al., *Antitick saliva antibody - a biologic marker of tick exposure that is a risk factor for Lyme disease seropositivity.* American Journal of Epidemiology, 1991. **134**(1): p. 86-95.
12. Orloski, K.A., et al., *Emergence of Lyme disease in Hunterdon County, New Jersey, 1993: a case-control study of risk factors and evaluation of reporting patterns.* American journal of epidemiology, 1998. **147**(4): p. 391-7.
13. Wallace, B.J., et al., *Human granulocytic ehrlichiosis in New York.* Archives of Internal Medicine, 1998. **158**(7): p. 769-773.
14. Townsend, A.K., R.S. Ostfeld, and K.H. Geher, *The effects of bird feeders on Lyme disease prevalence and density of Ixodes scapularis (Acari: Ixodidae) in a residential area of Dutchess County, New York.* Journal of Medical Entomology, 2003. **40**(4): p. 540-546.
15. Smith, P.F., et al., *Occupational risk of Lyme-disease in endemic areas of New York state.* Annals of the New York Academy of Sciences, 1988. **539**: p. 289-301.
16. Vaughn, M.F. and S.R. Meshnick, *Pilot Study Assessing the Effectiveness of Long-Lasting Permethrin-Impregnated Clothing for the Prevention of Tick Bites.* Vector-Borne and Zoonotic Diseases, 2011. **11**(7): p. 869-875.
17. Smith, G., et al., *Risk factors for Lyme disease in Chester County, Pennsylvania.* Public Health Reports, 2001. **116**(SUPPL. 1): p. 146-156.

18. Connally, N.P., H.S. Ginsberg, and T.N. Mather, *Assessing peridomestic entomological factors as predictors for Lyme disease.* Journal of Vector Ecology, 2006. **31**(2): p. 364-370.
19. Finch, C., et al., *Integrated assessment of behavioral and environmental risk factors for Lyme disease infection on Block Island, Rhode Island.* PLoS One, 2014. **9**(1): p. e84758.
20. Belongia, E.A., et al., *Tickborne Infections as a Cause of Nonspecific Febrile Illness in Wisconsin.* Clinical Infectious Diseases, 2001. **54449**.
