# Supplementary material for: Systematic review and meta-analysis of tick-borne disease risk factors in residential yards, neighborhoods, and beyond
Source: BMC Infect Dis. 2019 Oct 17;19:861. doi: 10.1186/s12879-019-4484-3 (PMC6798452; doi:10.1186/s12879-019-4484-3)
Supplement: Supplementary file 7 — Additional file 7: Table S5. Counts of measures at each spatial scale, relative to quartiles of Lyme disease incidence. Each count is the number of observations of disease risk factors at the specified spatial scale, and for studies in areas with Lyme disease incidence less than the quartile upper bound and greater than or equal to the upper bound of the previous quartile. Quartiles were computed based on the distribution of Lyme disease incidence in U.S. states (averaged over the years 2006-2016) and Canada (2009-2016). [file 12879_2019_4484_MOESM7_ESM.pdf]

Appendix Table A.5. Counts of measures at each spatial scale, relative to quartiles of Lyme disease incidence. Each count is the number of observations of disease risk factors at the specified spatial scale, and for studies in areas with Lyme disease incidence less than the quartile upper bound and greater than or equal to the upper bound of the previous quartile. Quartiles were computed based on the distribution of Lyme disease incidence in U.S. states (averaged over the years 2006-2016) and Canada (2009-2016).

Lyme disease incidence quartile	Yard count	Neighborhood count	Outside neighborhood count	Quartile: upper bound of Lyme disease incidence
1	1	1	2	17.827
2	6	1	10	29.227
3	1	0	0	36.582
4	51	3	22	58.636
