# Supplementary material for: Systematic review and meta-analysis of tick-borne disease risk factors in residential yards, neighborhoods, and beyond
Source: BMC Infect Dis. 2019 Oct 17;19:861. doi: 10.1186/s12879-019-4484-3 (PMC6798452; doi:10.1186/s12879-019-4484-3)
Supplement: Supplementary file 8 — Additional file 8: Table S6. Estimated odds ratio, standard error, 95% confidence interval, and P-values for effects of spatial scale and publication year on tick-borne disease risk, assuming neighborhood extends 750 m from property boundaries (Table S6.1) or 250 m from property boundaries (Table S6.2). [file 12879_2019_4484_MOESM8_ESM.pdf]

Table A.6.1. Estimated odds ratio, standard error, 95% confidence interval, and P-values for effects of spatial scale and publication year on tick-borne disease risk, assuming neighborhood extends 750 m from property boundaries.

spatial scale	odds ratio	SE	CI	P	number of studies	number of records
**Yard**	2.62	1.16	[1.97, 3.48]	<0.0001	13	59
**neighborhood**	4.1	1.29	[2.5, 6.72]	<0.0001	4	5
**outside neighborhood**	2.05	1.13	[1.61, 2.62]	<0.0001	8	33
**Year**	0.97	1.01	[0.96, 0.99]	<0.0001	NA	NA

Table A.6.2. Estimated odds ratio, standard error, 95% confidence interval, and P-values for effects of spatial scale and publication year on tick-borne disease risk, assuming neighborhood extends 250 m from property boundaries.

spatial scale	odds ratio	SE	CI	P	number of studies	number of records
**yard**	2.61	1.16	[1.96, 3.47]	<0.0001	13	59
**neighborhood**	4.08	1.29	[2.49, 6.69]	<0.0001	4	5
**outside neighborhood**	2.04	1.13	[1.6, 2.6]	<0.0001	9	34
**year**	0.97	1.01	[0.96, 0.99]	<0.0001	NA	NA
